# The effect of hypertension on cerebrovascular carbon dioxide reactivity in atrial fibrillation patients

**DOI:** 10.1038/s41440-024-01662-2

**Published:** 2024-04-10

**Authors:** Harvey J. Walsh, Rehan T. Junejo, Gregory Y. H. Lip, James P. Fisher

**Affiliations:** 1 Department of Physiology, Faculty of Medical & Health Sciences, University of Auckland, Auckland, New Zealand; 2 Department of Life Sciences, Faculty of Science and Engineering, Manchester Metropolitan University, Manchester, UK; 3 Liverpool Centre for Cardiovascular Science at University of Liverpool, Liverpool John Moores University and Liverpool Heart & Chest Hospital, Liverpool, UK; 4 Danish Center for Health Services Research, Department of Clinical Medicine, Aalborg University, Aalborg, Denmark

**Keywords:** Atrial fibrillation, Carbon dioxide, Cerebral blood flow, Hypertension

## Abstract

Atrial fibrillation (AF) and hypertension (HTN) are both associated with impaired cerebrovascular carbon dioxide reactivity (CVR_CO2_), an indicator of cerebral vasodilatory reserve. We hypothesised that CVR_CO2_ would be lower in patients with both AF and HTN (AF + HTN) compared to normotensive AF patients, due to an additive effect of AF and HTN on CVR_CO2_. Forty AF (68 ± 9 years) and fifty-seven AF + HTN (68 ± 8 years) patients underwent transcranial Doppler ultrasound measurement of middle cerebral artery blood velocity (MCA V_m_) during stepped increases and decreases in end-tidal carbon dioxide (P_ET_CO_2_). A cerebrovascular conductance index (CVCi) was calculated as the ratio of MCA V_m_ and mean arterial pressure (MAP). CVR_CO2_ was determined from the linear slope for MCA V_m_ and MCA CVCi vs P_ET_CO_2_. Baseline MAP was higher in AF + HTN than AF (107 ± 9 vs. 98 ± 9 mmHg, respectively; *p* < 0.001), while MCA V_m_ was not different (AF + HTN:49.6 [44.1–69.0]; AF:51.7 [45.2–63.3] cm.s^−1^; *p* = 0.075), and CVCi was lower in AF + HTN (0.46 [0.42–0.57] vs. 0.54 [0.44–0.63] cm.s^−1^.mmHg^−1^; p < 0.001). MCA V_m_ CVR_CO2_ was not different (AF + HTN: 1.70 [1.47–2.19]; AF 1.74 [1.54–2.52] cm/s/mmHg^−2^; *p* = 0.221), while CVCi CVR_CO2_ was 13% lower in AF + HTN (0.013 ± 0.004 vs 0.015 ± 0.005 cm.s^−1^.mmHg^−1^; p = 0.047). Our results demonstrate blunted cerebral vasodilatory reserve (determined as MCA CVCi CVR_CO2_) in AF + HTN compared to AF alone. This may implicate HTN as a driver of further cerebrovascular dysfunction in AF that may be important for the development of AF-related cerebrovascular events and downstream cognitive decline.

**Figure:**
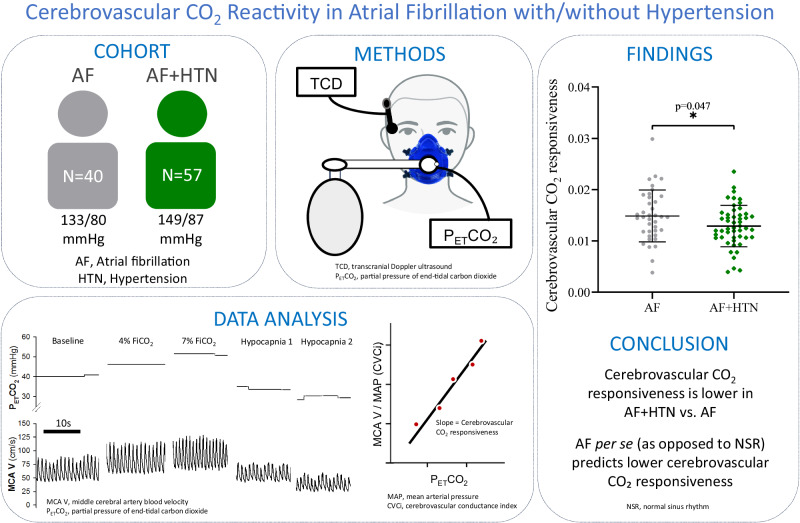
We demonstrated reduced cerebrovascular CO_2_ responsiveness in atrial fibrillation with hypertension (AF+HTN) vs. atrial fibrillation (AF). Furthermore, AF per se (as opposed to normal sinus rhythm) predicts reduced cerebrovascular CO_2_ responsiveness. Our findings suggest additional cerebrovascular dysfunction in AF+HTN vs. AF.

We demonstrated reduced cerebrovascular CO_2_ responsiveness in atrial fibrillation with hypertension (AF+HTN) vs. atrial fibrillation (AF). Furthermore, AF per se (as opposed to normal sinus rhythm) predicts reduced cerebrovascular CO_2_ responsiveness. Our findings suggest additional cerebrovascular dysfunction in AF+HTN vs. AF.

## Abstract (web summary)

We demonstrated reduced cerebrovascular CO_2_ responsiveness in atrial fibrillation with hypertension (AF+HTN) vs. atrial fibrillation (AF). Furthermore, AF per se (as opposed to normal sinus rhythm) predicts reduced cerebrovascular CO_2_ responsiveness. Our findings suggest additional cerebrovascular dysfunction in AF+HTN vs. AF.

## Introduction

Atrial fibrillation (AF) is a supraventricular arrhythmia affecting 0.5–1% of the global population [1], making it the most prevalent sustained cardiac rhythm disorder. Alarmingly, this number is projected to rapidly increase over the next 30 years as populations age and modern healthcare improves survival outcomes for acute cardiac events [1, 2].

Hypertension (HTN) is a powerful risk factor for AF, and has previously been reported in 40–90% of AF cohorts [3]. In addition, HTN is likely responsible for up to 50% of all AF diagnoses [3]. Downstream complications of AF include thromboembolic events such as stroke, of which AF patients are five times more likely to develop [4], and neurodegenerative disorders including cognitive decline and dementia, even in the absence of overt stroke [5]. Similarly, HTN is a known risk factor for stroke, present in ~60% of stroke occurrences [6], as well as cognitive impairment disorders [7]. Possible mechanisms for this phenomena include silent cerebral infarction [8], inflammation [9] and cerebral hypoperfusion [10].

In AF, emerging evidence suggests that impaired cerebrovascular function may also contribute to this heightened risk of stroke and cognitive decline. It is currently unknown if HTN exacerbates cerebrovascular dysfunction in AF. Peripheral vascular dysfunction is well-documented in AF. Endothelium-dependent brachial artery flow-mediated dilatation is reduced in AF patients and coupled with increased levels of plasma von Willebrand factor, a known biomarker of endothelial dysfunction [11]. Furthermore, increased oxidative stress [12] and the shift to a pro-inflammatory state [13] in AF patients synergistically worsen endothelial function. Moreover, hypertension evokes similar effects on peripheral vascular function. Structural vascular remodelling evoked by increased pressures and pulsatility increases oxidative stress and inflammation [14], causing vessel stiffening and subsequent endothelial dysfunction, as evidenced by reductions in nitric oxide (NO) production in HTN [15]. Interestingly, peripheral vascular function was previously reported to not be different between a group of HTN patients and a group with both AF and HTN (AF + HTN) [16], likely due to the shared vascular pathologies between AF and HTN. Evidence of cerebrovascular dysfunction also exists in AF. Cerebrovascular CO_2_ reactivity (CVR_CO2_), defined as the change in (∆) cerebral blood flow (CBF) versus ∆ partial pressure of CO_2_ (P_a_CO_2_), is widely recognized as a marker of cerebral vasodilatory reserve, and has been associated with increased risk of mortality when impaired [17]. AF patients exhibit worsened CVR_CO2_ compared to healthy controls, indicative of cerebrovascular dysfunction [18]. Furthermore, neurovascular coupling, whereby cerebral blood flow is increased to support neuronal metabolic needs, is reduced in AF [19]. CVR_CO2_ responses have also been reported to be worsened in hypertension [20] although this is not a universal finding [21]. The combined effects of AF and HTN on CVR_CO2_ are yet to be determined; thus, it is still unknown whether their effects on CVR_CO2_ are additive or occlusive when concurrently present.

The aim of this study was to assess whether AF patients with HTN exhibit worsened cerebral vasodilatory reserve (i.e., poorer CVR_CO2_) compared to normotensive AF patients. Considering the current evidence indicating the effects of AF and HTN independently on CVR_CO2_, we hypothesized that AF patients with hypertension would exhibit lowered CVR_CO2_ compared with normotensive AF patients.

## Methods

### Ethical approval

The National Research Ethics Service Committee West Midlands (15/WM/0447) and North West (17/NW/0714) approved all study procedures, all of which conformed with the Declaration of Helsinki. Prospective participants received an information sheet which they were verbally guided through in detail. After addressing any questions, participants provided written informed consent.

### Participant characteristics

Ninety-seven participants were included across two AF groups: AF (*n* = 40) and AF + HTN (*n* = 57). Of these, we included fifteen participants (which met the current inclusion criteria) from a previous study in which we used the same experimental approaches and outcome measures to cross-sectionally analyse cerebral vasodilatory reserve in three groups (AF, HTN and healthy controls) [18]. Patients were recruited from the following sites: Cardiology clinics at City Hospital, Birmingham, UK and Liverpool Heart and Chest Hospital, Liverpool, UK, along with National Institute of Health Research Clinical Research Network General Practitioner clinics in the UK.

All participants had previously been clinically diagnosed with AF and were included regardless of AF temporality (i.e. paroxysmal AF [fibrillation episodes are transient and spontaneously resolve within 48 h] or persistent AF [untreated fibrillation episodes last longer than 7 days before spontaneously resolving]). Study exclusion criteria included major illnesses such as cancer, liver or kidney disease, significant previous cardiovascular disease including myocardial infarction, left ventricular dysfunction, valvular heart disease, uncontrolled thyroid disorders, viral illness, connective tissue, inflammatory or neurological disease, significant cerebrovascular events such as stroke or transient ischaemic attack, alcohol abuse (>28 units per week), intravenous drug use or smoking, premenopausal women, use of oral nitrates or hormone replacement therapy and age <18 years. Tables 1 and 2 list participant characteristics and medication use respectively.

**Table 1:** Participant characteristics

	AF (*n* = 40)	AF + HTN (*n* = 57)	*P (d, φ)*
Age (years)	68 ± 9	68 ± 8	0.950
Women, *n* (%)	12 (30)	17 (30)	0.985
Weight (Kg)	79 [69–88]	88 [78–100]	0.004 (0.296)
Height (m)	1.73 ± 0.09	1.73 ± 0.10	0.822
BMI (kg.m^−2^)	26 [24–30]	30 [27–33]	<0.001 (0.355)
Waist (cm)	94 [84–101]	96 [94–105]	0.008 (0.290)
Hip (cm)	102 [99–109]	107 [103–116]	0.003 (0.327)
Waist / Hip	0.90 ± 0.08	0.90 ± 0.11	0.840
Waist / Height	0.54 ± 0.06	0.58 ± 0.06	0.007 (0.652)
Systolic BP (mmHg)	133 ± 16	149 ± 14	<0.001 (1.090)
Diastolic BP (mmHg)	80 ± 9	87 ± 12	0.001 (0.696)
MAP (mmHg)	98 ± 11	107 ± 9	<0.001 (0.908)
HR (b.min^−1^)	65 [58–70]	68 [61–81]	0.047 (0.202)
Fibrillating, *n* (%)	26 (67)	28 (49)	0.089
Persistent AF, *n* (%)	16 (52)	31 (61)	0.416
Diabetes, *n* (%)	1 (3)	4 (7)	0.646
CHA_2_DS_2_-VASc	1 [1-2]	2 [2–3]	<0.001 (0.372)
MoCA	26 ± 2	26 ± 2	0.889
Previous smoker, *n* (%)	16 (50)	27 (55)	0.653
Excessive alcohol consumption, *n* (%)	4 (10)	11 (19)	0.212

Mean ± SD displayed for normally distributed and median [interquartile range] for non-normally distributed continuous variables. Frequency (percentage) displayed for categorical discreate variables. Independent samples *t* test or Mann–Whitney U test used to infer statistical differences for continuous variables and Pearson’s or Fisher’s Exact Probability test for categorical data. Cohen’s *d* (*d*) and Phi (*φ)* reported as effect size for significant continuous and categorical variables, respectively. Patients in AF at the time of data acquisition are identified as ‘Fibrillating’. Significance: *P* ≤ 0.05

*AF* atrial fibrillation, *AF + HTN* atrial fibrillation with hypertension, *φ,* Phi, *BMI* body mass index, *BP* blood pressure, *HR* heart rate, *MAP* mean arterial pressure, *MoCA* Montreal Cognitive Assessment

**Table 2:** Cerebrovascular CO_2_ parameters

		AF	AF + HTN	*P (d)*
**MCA** V_m_ (cm.s^−1^.mmHg^−1^)	Linear	1.741 [1.543–2.517]	1.701 [1.472–2.186]	0.221
AF (n = 39), AF-HT (*n* = 51)	R^2^	0.952 [0.929–0.972]	0.952 [0.917–0.974]	0.533
	Exponent	0.031 [0.029–0.037]	0.032 [0.029–0.035]	0.919
	R^2^	0.973 [0.950–0.992]	0.976 [0.949–0.989]	0.347
**MCA CVCi** (cm.s^−1^.mmHg^−1^)	Linear	0.015 ± 0.005	0.013 ± 0.004	0.047 (0.434)
AF (*n* = 38), AF-HT (*n* = 50)	R^2^	0.957 [0.920–0.984]	0.947 [0.900–0.966]	0.140
	Exponent	0.027 ± 0.007	0.027 ± 0.006	0.851
	R^2^	0.957 [0.900–0.985]	0.957 [0.902–0.975]	0.674
**PCA** V_m_ (cm.s^−1^.mmHg^−1^)	Linear	1.076 ± 0.246	1.127 ± 0.352	0.587
AF (*n* = 19), AF-HT (*n* = 30)	R^2^	0.948 [0.919–0.979]	0.936 [0.876–0.967]	0.145
	Exponent	0.036 ± 0.011	0.035 ± 0.112	0.648
	R^2^	0.968 [0.921–0.987]	0.965 [0.911–0.986]	0.608
**PCA CVCi** (cm.s^−1^.mmHg^−1^)	Linear	0.008 ± 0.002	0.009 ± 0.003	0.892
AF (*n* = 20), AF-HT (*n* = 29)	R^2^	0.964 [0.899–0.988]	0.935 [0.878–0.978]	0.130
	Exponent	0.031 ± 0.010	0.031 ± 0.013	0.852
	R^2^	0.963 [0.885–0.986]	0.949 [0.863–0.967]	0.170

Mean ± SD displayed for normally distributed and median [interquartile range] for non-normally distributed variables. Independent samples t-test or Mann–Whitney U test used to infer statistical differences. Cohen’s *d* reported as effect size for significant variables. Significance: *p* ≤ 0.05

*AF* atrial fibrillation, *AF + HTN*, atrial fibrillation with hypertension, *CVCi* conductance index, *MCA* middle cerebral artery, *PCA* posterior cerebral artery, *V*_m_ mean velocity

### Experimental measures

A thorough participant history was recorded, including medication use, alcohol consumption and smoking habits. Using the recorded participant characteristics, each participant was calculated a CHA_2_DS_2_-VASc score. Anthropometric measures included height, weight, waist (umbilical level) and hip (femoral trochanter level) circumference. Non-invasive supine brachial BP was measured using an automated oscillometric device (M2, Omron, Kyoto, Japan). Beat-to-beat BP was continuously recorded by finger photoplethysmography (Finometer MIDI, Finapres Medical Systems, Amsterdam, The Netherlands), and heart rate (HR) by lead II ECG (BioAmp, ADInstruments, Dunedin, New Zealand). An oronasal mask connected to a heated pneumotach (Hans Rudolph, Kansas City, KS, USA) was used to record minute ventilation. A sampling line connected the oronasal mask to a capnograph (RespSense, Nonin Medical, Plymouth, MN, USA) for recording of end-tidal partial pressure of CO_2_ (P_ET_CO_2_). Bilateral 2 MHz transcranial Doppler probes (Doppler BosX, DWL, Sipplingen, Germany) were used to insonate the cerebral arteries fixed at their respective temporal windows, providing mean blood flow velocity (V_m_) for the left posterior cerebral artery (PCA) and the right middle cerebral artery (MCA). Insonation of the MCA was successful in all participants, but only achieved in forty-seven participants (48%) for the PCA.

### Experimental protocol

Pre-study guidelines included no strenuous exercise, food and caffeine intake for 12 h prior to their study, no alcohol the day before and day of their study and to refrain from consuming medication (excluding anticoagulants) on the morning of study. Physiological assessments were undertaken with participants lying supine on a bed, with their head on a pillow. After instrumentation, a 10-minute baseline period was acquired whilst participants breathed room air. During this baseline, the mean of 3 brachial BP recordings was used to correct beat-to-beat BP obtained by finger photoplethysmography. Stepped increases and decreases in P_ET_CO_2_ were then used to assess CVR_CO2_. Hypercapnic steps involved participants breathing through a Douglas bag circuit containing 4% CO_2_, followed by 7% CO_2_. Gas mixtures both contained 21% oxygen (N_2_ balanced). Each gas mixture was breathed for 4 min. Following hypercapnic steps, participants breathed room air to allow P_ET_CO_2_ to return to baseline. They were then required to alter their respiratory rate and depth to lower their P_ET_CO_2_ to an equal and opposite value to that achieved in the two hypercapnic steps. Each hypocapnic step was 2 minutes in duration. All participants completed the 4% hypercapnic step and its equal opposite hypocapnic step. Two participants did not complete the 7% hypercapnic step and one participant did not complete it’s equal opposite hypocapnic step. These participants were removed from our CVR_CO2_ analyses.

### Data analysis

The ratio of height and weight squared was used to express participants BMI. Waist to hip and waist to height ratios were calculated for each participant. A multi-channel data acquisition system (Powerlab 16/35 and Labchart Pro 8.1.13, ADInstruments) was used to record and digitize analogue signals at 1 KHz. HR was calculated on a beat-to-beat basis from the ECG. Mean arterial pressure (MAP) and was obtained by integrating the arterial BP waveform over the complete cardiac cycle on a beat-to-beat basis. Similarly, V_m_ was obtained by integrating the MCA and PCA V_m_ waveforms over the complete cardiac cycle on a beat-to-beat basis. Cerebrovascular conductance index (CVCi) was calculated as the ratio of MCA V_m_ or PCA V_m_ with MAP. CVCi was not calculated if either V_m_ or MAP values were determined to be statistical outliers. Baseline measures were averaged over the entire 10-minute duration. Hypercapnic and hypocapnic step change measures were obtained from the final minute of the experimental period. The linear slopes of MCA V_m_ and PCA V_m_ versus P_ET_CO_2_ across the range of hypo- and hypercapnic steps provided CVR_CO2_. Outliers were defined as ± interquartile range (IQR)*2.2 and excluded from analyses.

### Statistical analysis

Shapiro-Wilk test was used to assess data normality. Levene’s test was used to assess homogeneity of variance between group data. Independent two-tailed Students t-test was implemented to analyse normally distributed continuous data and Mann–Whitney U test for non-normally distributed continuous data. Fisher’s Exact Probability Test was used to analyse normally distributed categorical data and Pearson chi-square for non-normally distributed categorical data. Effect size for significant continuous variables reported with Cohen’s d (*d)* and Phi (*φ)* for significant categorical variables. Two-way analysis of variance (ANOVA) was implemented to compare the effect of patient group and P_ET_CO_2_ on MCA V_m_, MCA CVCi, PCA V_m_, PCA CVCi and MAP. For ANOVA, data normality was assessed by inspection of Q-Q plots for standardized residuals. Mauchly’s test of sphericity was used to assess the assumption of sphericity. If the assumption of sphericity was violated, a Greenhouse-Geisser correction was applied. Post-hoc pairwise comparisons were performed upon identification of significant main effects using t-test with Bonferroni correction. A forced entry regression model was used to determine the influence of variables of interest on baseline and exponential CVR_CO2_ parameters (MCA V_m_, MCA CVCi, PCA V_m_ and PCA CVCi). Multicollinearity and autocorrelation were tested to reduce the risk of regression model violations. Tolerance, variance inflation factor, and Durbin-Watson statistic were 0.570–0.950, 1.053–1.755 and 1.754–2.520 respectively. Mean ± standard deviation (SD) are presented for normally distributed data, median [interquartile range] for non-normally distributed data and frequency (percentage) for categorical data. Statistical significance was considered as *p* < 0.05. Analysis was performed using SPSS, version 27 (IBM Corp., Armonk, NY, USA).

## Results

### Participant characteristics

Age and the proportion of males and females was not different in the AF and AF + HTN groups (Table 1). AF + HTN exhibited greater systolic BP (*p* < 0.001), diastolic BP (*p* = 0.001), MAP (*p* < 0.001), HR (*p* = 0.047), weight (*p* = 0.004), BMI (*p* < 0.001), waist (*p* = 0.008) and hip (*p* = 0.003) measurements, waist-to-height ratio (*p* = 0.007), and CHA_2_DS_2_-VASc scores (*p* < 0.001). The proportion of participants who were fibrillating at the time of the study, had a diagnosis of persistent AF, were diabetic, had previously smoked or previously consumed excessive alcohol ( > 14 units a week) were not different between AF and AF + HTN groups (*p* > 0.05). AF + HTN had more frequent ACE inhibitor (*p* < 0.001), Ca^2+^ channel inhibitor (*p* < 0.001), ARB (*p* < 0.001), statin (*p* = 0.017) and SSR inhibitor (*p* = 0.018) use (Supplementary Table 1).

### Baseline cerebral hemodynamics

**Fig. 1:**
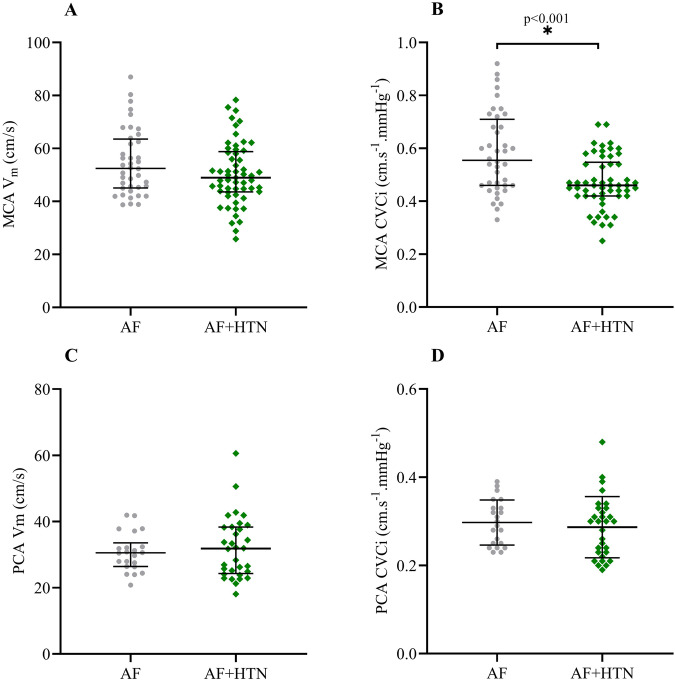
Baseline MCA V_m_ (**A**), MCA CVCi (**B**), PCA V_m_ (**C**), and PCA CVCi (**D**) in participants with AF or AF + HTN. Individual values plotted with bars displaying median and interquartile range for MCA V_m_ MCA CVCi and PCA V_m_, and mean and standard deviation for PCA CVCi. AF atrial fibrillation; AF + HTN atrial fibrillation with hypertension; MCA V_m_ middle cerebral artery mean blood velocity; PCA V_m_ posterior cerebral artery mean blood velocity; MCA CVCi middle cerebral artery cerebrovascular conductance index; PCA CVCi posterior cerebral artery cerebrovascular conductance index

Figure 1 shows baseline cerebrovascular measures. MCA V_m_ was numerically lower in AF + HTN (49.6 [44.1–60.0] cm.s^−1^) compared to AF (51.7 [45.2–63.3] cm.s^−1^), but did not reach statistical significance (*p* = 0.075). MCA CVCi was lower in AF + HTN (0.46 [0.42–0.55] cm.s^−1^.mmHg^−1^) compared to AF (0.56 [0.46–0.71] cm.s^−1^.mmHg^−1^; *p* < 0.001). No differences were observed between groups for either PCA V_m_ (AF: 30.5 [26.4–33.6]; AF + HTN: 31.8 [24.3–38.4] cm.s^−1^; *p* = 0.731) or PCA CVCi (AF: 0.30 ± 0.05; AF + HTN: 0.29 ± 0.07 cm.s^−1^.mmHg^−1^; *p* = 0.548). Baseline P_ET_CO_2_ was not different between groups (AF: 39.8 ± 0.7 mmHg; AF + HTN: 40.1 ± 0.7 mmHg; *p* = 0.813).

### Cerebrovascular reactivity

MCA V_m_, MCA CVCi, PCA V_m_, and PCA CVCi increased progressively with each stepped increase in P_ET_CO_2_ (all *p* < 0.001). MAP increased during the 7% hypercapnic step versus all other steps (all *p* < 0.001). MCA CVCi and MAP were lower in AF + HTN than AF (group effect, *p* < 0.001 and *p* = 0.002, respectively), while MCA V_m_, PCA V_m_ and PCA CVCi were not different between AF and AF + HTN groups (all *p* > 0.05). No interaction was observed between group and P_ET_CO_2_ for any variable examined (*p* > 0.05), although there was a strong tendency (*p* = 0.083) for an interaction between group and P_ET_CO_2_ for MCA CVCi (Supplementary Fig. 1).

**Fig. 2:**
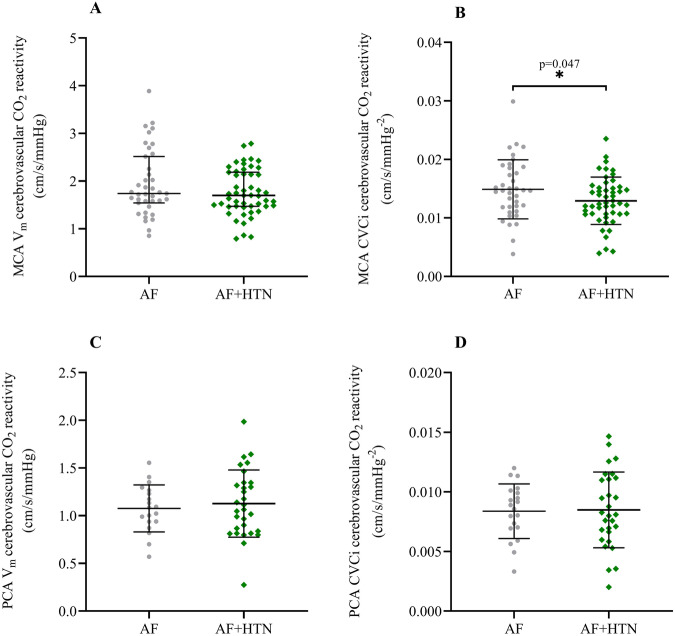
MCA V_m_ (**A**), MCA CVCi (**B**), PCA V_m_ (**C**) and PCA CVCi (**D**) CVR_CO2_ in participants with AF or AF + HTN. CO_2_ reactivity is expressed as the change in (∆) MCA V_m_, MCA CVCi, PCA V_m_ and PCA CVCi versus ∆ P_ET_CO_2_ in mmHg. Bars display median and interquartile range for MCA V_m_ CVR_CO2_, and mean and standard deviation for MCA CVCi, PCA V_m_ and PCA CVCi CVR_CO2_. AF atrial fibrillation; AF + HTN atrial fibrillation with hypertension; CVR_CO2_ cerebrovascular carbon dioxide reactivity; MCA V_m_ middle cerebral artery mean blood velocity; PCA V_m_ posterior cerebral artery mean blood velocity; MCA CVCi middle cerebral artery cerebrovascular conductance index; PCA CVCi posterior cerebral artery cerebrovascular conductance index; P_ET_CO_2_ partial pressure of end-tidal carbon dioxide

Indices of CVR_CO2_ are displayed in Table 2 and Fig. 2. MCA CVCi CVR_CO2_ was significantly lower in AF + HTN (0.013 ± 0.004 cm/s/mmHg^−2^) compared to AF (0.015 ± 0.005 cm/s/mmHg^−2^; *p* = 0.047). However, MCA V_m_ CVR_CO2_ (AF: 1.741 [1.543–2.517]; AF + HTN: 1.701 [1.472–2.186] cm/s/mmHg^−2^; *p* = 0.221), PCA V_m_ (AF: 1.076 ± 0.246; AF + HTN: 1.127 ± 0.352 cm/s/mmHg^−2^; *p* = 0.587) and PCA CVCi (AF: 0.008 ± 0.002; AF + HTN: 0.009 ± 0.003 cm/s/mmHg^−2^; *p* = 0.892) were not different between groups.

### Forced entry regression

Significant predictors of baseline variables and CVR_CO2_ as identified from forced entry linear regression are shown in Table 3. Fibrillation (vs normal sinus rhythm [NSR]) at the time of the experimental visit was associated with a lower MCA V_m_ CVR_CO2_. The presence of hypertension was associated with a lower baseline MCA CVCi, and being female was associated with higher baseline MCA V_m_ and baseline MCA CVCi. None of our entered independent variables were found to be associated with baseline PCA V_m_, baseline PCA CVCi, MCA CVCi CVR_CO2_, PCA V_m_ CVR_CO2_ and PCA CVCi CVR_CO2_.

**Table 3:** Forced entry multiple regression with baseline MCA V_m_ (cm∙s^−1^), baseline MCA CVCi (cm.s^−1^.mmHg^−1^), baseline PCA V_m_ (cm∙s^−1^), baseline PCA CVCi (cm.s^−1^.mmHg^−1^), MCA V_m_ (cm.s^−1^.mmHg^−1^), MCA CVCi (cm.s^−1^.mmHg^−1^), PCA V_m_ (cm.s^−1^.mmHg^−1^) and PCA CVCi (cm.s^−1^.mmHg^−1^) as dependent variables

Dependent variables	β	95% CI for β	*p*
Baseline MCA V_m_ (cm∙s^−1^)			
R = 0.472; R2 = 0.223; Adjusted R2 = 0.126; *p* = 0.050			
Sex	0.297	0.915–14.843	0.027
Baseline MCA CVCi (cm.s^−1^.mmHg^−1^)			
R = 0.590; R2 = 0.348; Adjusted R2 = 0.265; *p* = 0.002			
Hypertension	−0.373	−0.169–0.036	0.003
Sex	0.302	0.017–0.161	0.016
Baseline PCA V_m_ (cm.s^−1^)			
R = 0.334; R2 = 0.111; Adjusted R2 = −0.094; *p* = 0.770			
Baseline PCA CVCi (cm.s^−1^.mmHg^−1^)			
R = 0.315; R2 = 0.099; Adjusted R2 = −0.117; *p* = 0.831			
MCA V_m_ CVR_CO2_ (cm.s^−1^.mmHg^−1^)			
R = 0.459; R2 = 0.211; Adjusted R2 = 0.108; *p* = 0.078			
Fibrillating	−0.466	−0.965–0.138	0.010
MCA CVCi CVR_CO2_ (cm.s^−1^.mmHg^−1^)			
R = 0.419; R2 = 0.176; Adjusted R2 = 0.068; *p* = 0.159			
PCA V_m_ CVR_CO2_ (cm.s^−1^.mmHg^−1^)			
R = 0.405; R2 = 0.164; Adjusted R2 = −0.037; *p* = 0.568			
PCA CVCi CVR_CO2_ (cm.s^−1^.mmHg^−1^)			
R = 0.297; R2 = 0.088; Adjusted R2 = −0.140; *p* = 0.880			

Age, sex, cardiac rhythm at the time of data acquisition (AF versus normal sinus rhythm), type of AF (i.e. paroxysmal versus persistent), years since AF diagnosis, and hypertension status (yes/no) variables were entered into the model. The predictor variable ‘Fibrillating” indicates AF cardiac rhythm (as opposed to normal sinus rhythm) during data acquisition

*α* alpha, *β* beta, *CI* confidence interval, *CVCi* cerebrovascular conductance index, *MAP* mean arterial pressure, *MCA* middle cerebral artery, *PCA* posterior cerebral artery, *V*_m_ mean velocity, *AF* atrial fibrillation

## Discussion

This is the first study to investigate whether the presence of HTN as a comorbidity in AF worsens CVR_CO2_. The major novel finding of the present study is that CVCi CVR_CO2_ is lowered in AF + HTN compared to AF, and may implicate an additional level of cerebrovascular dysfunction in AF + HTN. This may be important for the development of cerebrovascular events and downstream cognitive decline commonly associated with AF.

Baseline MCA V_m_ was not different between AF and AF + HTN groups, indicating that cerebral perfusion at rest is retained in AF + HTN similar to that in AF. It is well accepted that there is a rightward shift in the cerebral autoregulatory curve in HTN, whereby CBF is maintained similarly to that in normotensives with higher MAP [22]. This may explain our conflicting baseline responses in terms of MCA Vm and CVCi. As a consequence of increased baseline MAP in AF + HTN, MCA CVCi is lowered, in order to buffer the transmission of peripheral BP to CBF. This may be explained due to HTN evoked remodelling, hypertrophy and stiffening within the cerebral arteries and arterioles, resulting in increased wall-lumen ratios and decreased vessel diameters [23]. These effects on the vasculature may be explained by a number of interconnected processes. Oxidative stress is heightened in HTN, and promotes proliferation of vascular smooth muscle cells as well as extracellular matrix remodelling [14]. Elevated reactive oxygen species (ROS) can also drive inflammation in HTN, resulting in further vascular hypertrophy. Indeed, elevated markers of inflammation, including C-reactive protein and interleukin-6, are independently associated with HTN [24]. Furthermore, the direct mechanical effects of heightened pressure on vessel walls can promote hypertrophy and remodelling through activation of extracellular matrix protein cascades, whereby noncellular material accumulates within vessel walls [25]. This can also drive ROS production, resulting in a pathological cycle of cerebrovascular remodelling.

Interestingly, in the present study we have shown a reduction in MCA CVCi CVR_CO2_ in AF + HTN but no difference in MCA V_m_ CVR_CO2_ between AF and AF + HTN. This offers distinct insight from our previous work, where we compared CVR_CO2_ in separate groups with either AF, HTN, or healthy controls (i.e. normotensive and with a normal sinus rhythm), and demonstrated lower MCA V_m_ CVR_CO2_ in AF versus HTN and healthy controls. The current study, in which all patients studied had AF, suggests that in AF + HTN, CO_2_-mediated increases in CBF per unit pressure are blunted. The use of a conductance index allows us to somewhat account for changes in perfusion pressure and provides more insight to vasodilator responsiveness. Lowered CVCi CVR_CO2_ may be indicative of additional cerebrovascular dysfunction evoked by HTN. The aforementioned HTN induced cerebrovascular remodelling will have functional consequences in terms of vascular responsiveness to CO_2_ that may drive differences in CVCi CVR_CO2_ between our AF + HTN and AF groups. In addition, endothelial damage/dysfunction associated with oxidative stress, inflammation, and reductions in NO bioavailability [26] and known to be present in both HTN and AF may also contribute and summate to worsen CVCi CVR_CO2_ in AF + HTN. In contrast, brachial artery flow mediated dilatation, a marker of peripheral endothelial function, is not different in HTN versus AF + HTN [16], and might suggest a stronger contribution of HTN than AF to peripheral/cerebrovascular dysfunction [22].

Forced entry regression suggested patients that were fibrillating at the time of study (as opposed to being in NSR) exhibited lower MCA V_m_ CVR_CO2_. MRI and xenon inhalation studies have previously shown improved CBF in AF patients who underwent NSR restoration procedures [27, 28], suggesting a direct effect of fibrillation on cerebral perfusion per se. However, in earlier work [18] we observed that MCA V_m_ was lower in AF patients that were fibrillating at the time of study compared to those in NSR, but MCA V_m_ CVR_CO2_ was not different. Differences in sample size (n = 97 in the present study vs. n = 31 in Junejo et al. [18]) and the analytical approach employed, may partly explain the discrepant findings, and further work is required to better understand how AF per se affects CVR_CO2_ and by what mechanisms.

The anterior and posterior cerebral circulations differ in regards to CVR_CO2_, with the posterior cerebral circulation exhibiting absolute CVR_CO2_ values ~ 50% lower than that seen in the anterior cerebral circulations [29, 30], which our results generally agree with. Additionally, differences may exist in endothelial function between the two circulations, as endothelium-derived NO has been shown to play a greater role in vasodilatory responses in the anterior cerebral circulation [31]. Notably, baseline PCA and PCA CVR_CO2_ measures were not different between AF and AF + HTN. This may indicate preserved endothelial function in the posterior cerebral circulation in AF + HTN vs. AF groups.

Our findings should be interpreted in light of several experimental considerations. While transcranial Doppler ultrasound offers real-time measurements of cerebrovascular function, is non-invasive and easy to implement during physiological interventions, its inability to directly measure blood flow is a limitation. Vessel diameter cannot be measured with transcranial Doppler, thus changes in cerebral blood velocity may not be directly indicative of CBF changes. However, as transcranial Doppler-derived V_m_ changes have been significantly correlated with gold standard measures of CBF (i.e., gadolinium tracer MRI and arterial spin labelling MRI [32]) V_m_ values are generally considered proportional to CBF. P_ET_CO_2_ has a strong positive correlation with P_a_CO_2_ [33], and acts as a reliable non-invasive surrogate measure, though we acknowledge its possible underestimation of P_a_CO_2_ at rest [34]. There is debate surrounding the optimal method for assessing cerebrovascular function [35]. The advantages of using fixed concentrations of CO_2_ (4 and 7%) as in the present study, is that the experimental set-up is relatively simple, inexpensive, convenient in the clinic, does not required a complex computerised gas delivery system, and requires minimal participant cooperation. This method has also shown good between-day test-retest reliability [18]. Our primary measure of cerebrovascular responsiveness (i.e., CVCi CVR_CO2_) was derived from the linear slope of the relationship between P_ET_CO_2_ and MCA CVCi for each patient and was significantly (p = 0.047) lower in AF + HTN versus AF. While a tendency (p = 0.083) for an interaction between Group and P_ET_CO_2_ was observed for MCA CVCi (Supplementary Fig. 1B) this did not reach significance. This discrepancy is likely caused by differences in the methods of data analysis used. We did not screen for the presence of subclinical brain infarcts, alhough patient history and clinical records did not suggest any presence of these. In addition, although participants were requested to refrain from consuming medications on the morning prior to their study visit, it is unlikely that this was sufficient to cause full washout prior to data acquisition. Finally, it is a limitation of the current study that a healthy control group (i.e., normotensive and NSR) was not recruited, however we believe that this does not diminish our ability to achieve the primary aim of the study which was to determine whether the presence of HTN in AF worsens cerebrovascular responsiveness.

In conclusion, this is the first study that demonstrates reduced CVR_CO2_ in AF patients with concurrent HTN as compared to normotensive AF patients. This may be important for clinical management due to the possible heightened risk of cerebrovascular events and downstream cognitive decline.

### Supplementary information

Supplementary figure and table

## Data Availability

Study data are available from the corresponding author upon reasonable request.
