# Supplementary material for: The effect of hypertension on cerebrovascular carbon dioxide reactivity in atrial fibrillation patients
Source: Hypertens Res. 2024 Apr 10;47(6):1678–87. doi: 10.1038/s41440-024-01662-2 (PMC11150149; doi:10.1038/s41440-024-01662-2)
Supplement: Supplementary file 1 — Supplementary figure and table [file 41440_2024_1662_MOESM1_ESM.docx]

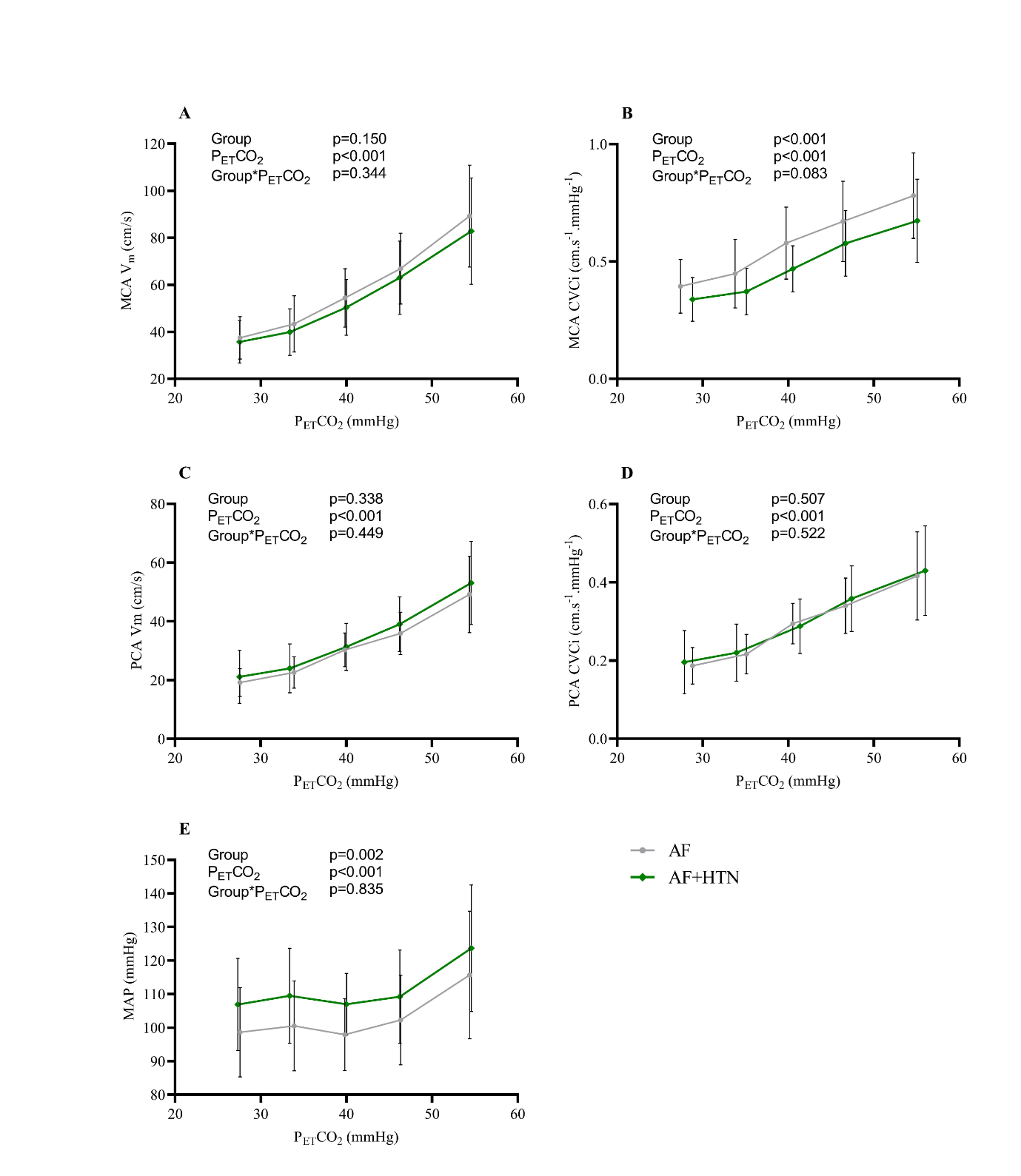
**Supplementary Figure 1. MCA V_m_ (A), MCA CVCi (B), PCA V_m_ (C), PCA CVCi (D) and MAP (E) responses to P_ET_CO_2_ changes in participants with AF or AF+HTN.**

Mean values plotted with bars displaying standard deviation. Group, P_ET_CO_2_ and interaction terms as determined by two-way repeated measures ANOVA displayed on plots. AF, atrial fibrillation; AF+HTN, atrial fibrillation with hypertension; ANOVA, analysis of variance; MCA V_m_, middle cerebral artery mean blood velocity; PCA V_m_, posterior cerebral artery mean blood velocity; MCA CVCi, middle cerebral artery cerebrovascular conductance index; PCA CVCi, posterior cerebral artery cerebrovascular conductance index; P_ET_CO_2_, partial pressure of end-tidal carbon dioxide.

**Supplementary Table 1.** Participant medication use.

|  | AF (n = 40) | AF+HTN (n = 57) | *p (φ)* |
| --- | --- | --- | --- |
| Anticoagulants, *n* (%) | 36 (90) | 54 (95) | 0.442 |
| α inhibitor, *n* (%) | 3 (8) | 3 (5) | 0.688 |
| β inhibitor, *n* (%) | 19 (48) | 35 (61) | 0.175 |
| Cardiac glycoside, *n* (%) | 3 (8) | 11 (19) | 0.104 |
| Anti-arrhythmic, *n* (%) | 7 (18) | 4 (7) | 0.191 |
| ACE inhibitor, *n* (%) | 1 (3) | 23 (40) | <0.001 (0.432) |
| Ca^2+^ channel inhibitor, *n* (%) | 0 (0) | 21 (37) | 0.000 (0.440) |
| ARB, *n* (%) | 0 (0) | 15 (26) | 0.000 (0.358) |
| Loop diuretic, *n* (%) | 0 (0) | 4 (7) | 0.140 |
| Thiazide diuretic, *n* (%) | 0 (0) | 3 (5) | 0.265 |
| Biguanide, *n* (%) | 1 (3) | 3 (5) | 0.641 |
| PP inhibitor, *n* (%) | 5 (13) | 14 (25) | 0.141 |
| Statin, *n* (%) | 14 (35) | 34 (60) | 0.017 (0.243) |
| SSR inhibitor, *n* (%) | 6 (15) | 1 (2) | 0.018 (-0.252) |
| Tricyclic antidepressant, *n* (%) | 0 (0) | 2 (4) | 0.510 |
| SNR inhibitor, *n* (%) | 0 | 1 (2) | 1.000 |
| NSAIDs, *n* (%) | 6 (15) | 3 (5) | 0.155 |
| Folic acid, *n* (%)  Opioids, *n* (%)  β2 agonists, *n* (%)  Sulfasalazine, *n* (%)  Xanthine Oxidase Inhibitor, *n* (%)  5-α reductase inhibitors, *n* (%)  Dopamine antagonist, *n* (%)  Thyroxine, *n* (%)  Anticonvulsants, *n* (%)  Muscarinic antagonist, *n* (%)  Vitamin D, *n* (%)  Anti-histamine, *n* (%)  Mirtazipine, *n* (%)  Nitrazepam, *n* (%)  Erythromycin, *n* (%)  Rantidine, *n* (%)  Paracetamol, *n* (%)  Musculotropic antispasmodic, *n* (%)  Insulin, *n* (%) | 0  1 (3)  0 (0)  1 (3)  0 (0)  1 (3)  0 (0)  2 (5)  0 (0)  1 (3)  2 (5)  1 (3)  1 (3)  1 (3)  1 (3)  1 (3)  1 (3)  0 (0)  0 (0) | 1 (2)  1 (2)  1 (2)  0 (0)  4 (7)  1 (2)  1 (2)  7 (12)  3 (5)  2 (4)  3 (5)  1 (2)  0 (0)  0 (0)  0 (0)  0 (0)  0 (0)  2 (4)  1 (2) | 1.000  1.000  1.000  0.412  0.140  1.000  1.000  0.300  0.265  1.000  1.000  1.000  0.412  0.412  0.412  0.412  0.412  0.510  1.000 |

Frequency (percentage) displayed for all values. Pearson’s or Fisher’s Exact Probability test used to test for statistical differences. Phi reported as effect size. Significance: *p* ≤ 0.05. α, Alpha; β, Beta, φ, Phi; ACE, angiotensin converting enzyme; AF, atrial fibrillation; AF+HTN, atrial fibrillation with hypertension; ARB, angiotensin receptor blocker; BMI, body mass index; Ca^2+^, calcium; NSAIDs, non-steroidal anti-inflammatory drugs; PPI, proton pump; SNR, serotonin noradrenaline reuptake; SSR, selective serotonin reuptake.
